# The Probiotic Strains *Bifidοbacterium lactis, Lactobacillus acidophilus,* *Lactiplantibacillus plantarum* and *Saccharomyces boulardii* Regulate Wound Healing and Chemokine Responses in Human Intestinal Subepithelial Myofibroblasts

**DOI:** 10.3390/ph15101293

**Published:** 2022-10-20

**Authors:** Gesthimani Tarapatzi, Eirini Filidou, Leonidas Kandilogiannakis, Michail Spathakis, Maria Gaitanidou, Konstantinos Arvanitidis, Ioannis Drygiannakis, Vassilis Valatas, Katerina Kotzampassi, Vangelis G. Manolopoulos, George Kolios, Stergios Vradelis

**Affiliations:** 1 Laboratory of Pharmacology, Faculty of Medicine, Democritus University of Thrace, 68100 Alexandroupolis, Greece; 2 Individualised Medicine & Pharmacological Research Solutions Center (IMPReS), 68100 Alexandroupolis, Greece; 3 Gastroenterology and Hepatology Research Laboratory, Medical School, University of Crete, 71003 Heraklion, Greece; 4 Department of Surgery, Aristotle University of Thessaloniki, 54636 Thessaloniki, Greece; 5 Second Department of Internal Medicine, University Hospital of Alexandroupolis, Democritus University of Thrace, 68100 Alexandroupolis, Greece

**Keywords:** probiotics, chemokines, wound healing, human intestinal subepithelial myofibroblasts

## Abstract

*Bifidobacterium lactis*, *Lactobacillus acidophilus, Lactiplantibacillus plantarum* and *Saccharomyces boulardii* are common probiotic supplements. Colonic subepithelial myofibroblasts (cSEMFs) are actively involved in mucosal wound healing and inflammation. cSEMFs, isolated from healthy individuals, were stimulated with 10^2^ or 10^4^ cfu/mL of these probiotic strains alone and in combination, and their effect on chemokine and wound healing factor expression was assessed by qRT-PCR, ELISA and Sircol Assay, and on cSEMFs migration, by Wound Healing Assay. These strains remained viable and altered cSEMFs’ inflammatory and wound healing behavior, depending on the strain and concentration. cSEMFs treated with a combination of the four probiotics had a moderate, but statistically significant, increase in the mRNA and/or protein expression of chemokines CXCL1, CXCL2, CXCL4, CXCL8, CXCL10, CCL2 and CCL5, and healing factors, collagen type I and III, fibronectin and tissue factor. In contrast, when each strain was administered alone, different effects were observed, with greater increase or decrease in chemokine and healing factor expression, which was balanced by the mixture. Overall, this study highlights that the use of multiple probiotic strains can potentially alert the gut mucosal immune system and promote wound healing, having a better effect on mucosal immunity than the use of single probiotics.

## 1. Introduction

Microbial dysbiosis in the gut, i.e., the disturbed coexistence of the various bacteria and fungi alongside the human cells, could be both the cause and the result of a wide range of inflammatory diseases [1,2]. Since the importance of the interplay between the gut and its microflora has been highlighted, more and more probiotic supplements have been implemented in auxiliary treatments, due to their ability to not only ameliorate intestinal inflammation [3,4], but also to promote mucosal healing [5]. However, which probiotic strains are the most helpful and in which concentrations and conditions needs yet to be elucidated. 

Bacteria of the genus *Lactobacillus* have been widely known for their anti-inflammatory effects [6,7], with *Lactiplantibacillus plantarum* (previously known as *Lactobacillus plantarum*) being an important regulator of Th1, Th2 and Treg immunity cytokine pathways. Specifically, this bacterial strain and its metabolome mediate the production of both anti-inflammatory IL-10 and pro-inflammatory cytokines, such as IFN-γ, IL-1β, IL-2, IL-6, IL-10, IL-12, IL-17 and TNF-α [8,9,10,11,12,13,14]. Heat-inactivated or live *L. plantarum* or its supernatants could also promote skin wound healing, while simultaneously downregulating fibrotic disposition [15,16,17].

In addition, another *Lactobacillus* strain, *Lactobacillus acidophilus,* plays a key role in gut homeostasis, mainly through alleviating re-epithelialization and regulating Th17 and Treg immunological cascades [18,19,20,21,22]. Regarding *L. acidophilus’* impact on healing, it seems to be commonly used among probiotic mixes with other lactic acid bacteria [23,24], but it is not adequately studied when administered alone, apart from a study by Bahr et al., where it downregulated the expression of TGF-β and α-SMA [25].

*Bifidobacterium lactis’* role is important in ameliorating the pro-inflammatory processes of the intestine, mainly contributing to the reduction in factors such as IFN-γ, IL-1β, IL-6, IL-8, IL-17 and TNF-α [26,27,28,29,30,31]. Huang et al. and Turner et al. showed that pretreatment with this strain can also benefit the innate immunity responses towards pathogen infections [32,33]. Furthermore, *B. lactis* is able to strengthen the gut barrier, by alleviating the production of tight junction proteins, such as Claudin-1, MUC2, Occludin and ZO-1 [31,34,35].

The probiotic fungus *Saccharomyces boulardii* can act as an anti-fibrotic and anti-inflammatory mediator. On the one hand, as the aforementioned probiotics, it can mediate inflammatory signals through upregulating IL-10 and downregulating IFN-γ, IL-1β, IL-6 and TNF-α [36,37,38,39,40,41,42]. On the other hand, it can contribute to the integrity of the intestinal barrier by inducing the repair of the epithelial cells [42,43,44,45]. Last but not least, *S. boulardii* supplementation can reduce fibrotic factors, including collagen type I, TGF-β and α-SMA [36].

Colonic subepithelial myofibroblasts (cSEMFs) are stellate-shaped cells, residing within the lamina propria of the gut and expressing α-smooth muscle actin and vimentin [46,47]. cSEMFs are key components of intestinal integrity and wound healing, due to their ability to migrate to the trauma region, excrete extracellular matrix proteins, such as collagen, and support the epithelial regeneration [47,48,49]. In addition, these cells both express a wide range of cytokine receptors and produce immunological mediators and growth factors, for instance TGF-β, and are therefore involved not only in wound re-epithelization but also in the inflammatory and fibrotic cascades of the gut [47,50,51]. In DSS-induced colitis in mice, treatment with *Lactiplantibacillus plantarum* has shown a myofibroblast-mediated anti-inflammatory and anti-fibrotic effect [52], while in another in vitro study utilizing human myofibroblasts, a mix of eight different probiotic bacteria, including *Lactobacillus* and *Bifidobacterium* strains, has proven efficacy in TGF-β inhibition [53]. Apart from offering their innate properties, i.e., the regulation of PGE_2_ production by cultured myofibroblasts stimulated with *Lactobacillus rhamnosus* [54] and intestinal organoids stimulated with *Lactobacillus acidophilus* [55], recombinant probiotics could even be used as regulatory protein domain carriers [56].

Although there are many anti-inflammatory and anti-fibrotic advances in using different combinations of bacterial and fungal strains, there is still the need to identify the key interactions between the microflora and the human host, as well as their implication both in symbiosis and the pathology of intestinal conditions. In this study, we aim to investigate the effects of the probiotic strains *Bifidοbacterium lactis, Lactobacillus acidophilus, Lactiplantibacillus plantarum* and *Saccharomyces boulardii*, alone and in combination, on the expression of chemokines and wound-healing-related factors and on the migratory rate of human colonic subepithelial myofibroblasts.

## 2. Results

### 2.1. Viability of the Probiotic Strains

**Figure 1:**
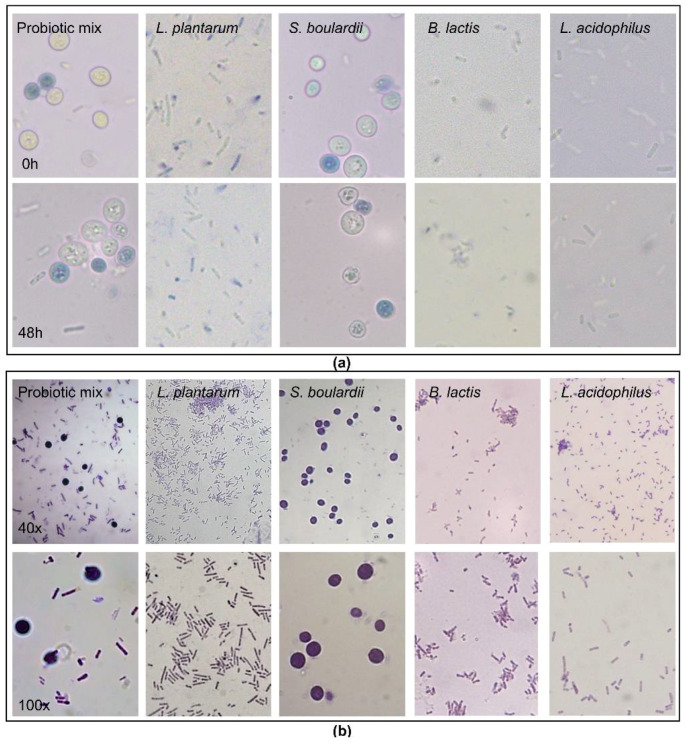
(**a**) Optical representation of Trypan Blue staining of the Probiotic mix, *Lactiplantibacillus plantarum*, *Saccharomyces boulardii*, *Bifidobacterium lactis* and *Lactobacillus acidophilus* at the time of dilution (0 h) and after 48 h incubation. (**b**) Gram staining of the Probiotic mix, *Lactiplantibacillus plantarum*, *Saccharomyces boulardii*, *Bifidobacterium lactis* and *Lactobacillus acidophilus* using 40× and 100× magnification.

Using Trypan Blue dye, we confirmed that both the probiotic mix of *Bifidobacterium lactis, Lactobacillus acidophillus, Lactiplantibacillus plantarum* and *Saccharomyces boulardii* and each strain alone remained viable (white cells) in the cSEMF culture medium after a 48 h incubation (Figure 1a), which correlates with the time period of the rest of the experimental procedures of the current study.

### 2.2. Gram Staining of the Probiotic Strains

We further proceeded to a visualization of the probiotics, with Gram staining. As it was already known, all four strains were confirmed as Gram-positive (Figure 1b).

### 2.3. Probiotic Stimulation Affects cSEMF Responses

#### 2.3.1. The Probiotic Strains Regulate the Chemokine mRNA Expression on cSEMFs

**Figure 2:**
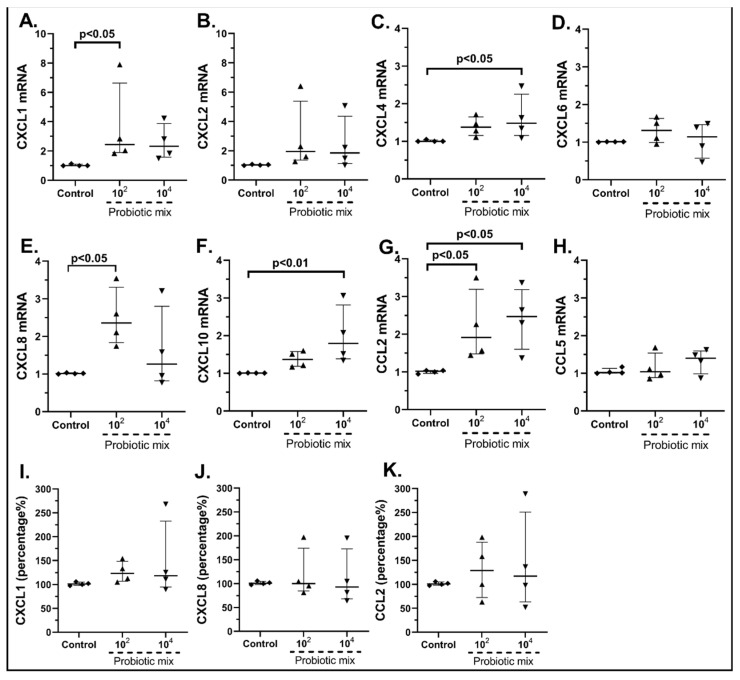
cSEMF mRNA expression of CXCL1 (**A**), CXCL2 (**B**), CXCL4 (**C**), CXCL6 (**D**), CXCL8 (**E**), CXCL10 (**F**), CCL2 (**G**) and CCL5 (**H**) and protein levels of CXCL1 (**I**), CXCL8 (**J**) and CCL2 (**K**) after stimulation with the probiotic mix of *Bifidobacterium lactis, Lactobacillus acidophilus, Lactiplantibacillus plantarum* and *Saccharomyces boulardii.* Results are presented as median with interquartile range. N = 4.

Unstimulated cSEMFs had a baseline expression of all the studied chemokines. The effect of the probiotic mix of *Bifidobacterium lactis, Lactobacillus acidophilus, Lactiplantibacillus plantarum* and *Saccharomyces boulardii* on cSEMF chemokine production was different, depending on the concentration used. More specifically, the stimulation of cSEMFs with 10^2^ cfu/mL resulted in the upregulation of the mRNAs of CXCL1 (2.44-fold, IQR: 1.89–6.63, *p* < 0.05, Figure 2A) and CXCL8 (2.35-fold, IQR: 1.83–3.31, *p* < 0.001, Figure 2E), while stimulation with 10^4^ cfu/mL upregulated the mRNA expression of CXCL4 (1.48-fold, IQR: 1.15–2.25, *p* < 0.05, Figure 2C) and CXCL10 (1.79-fold, IQR: 1.39–2.81, *p* < 0.01, Figure 2F). In addition, both concentrations upregulated the mRNA of CCL2 (10^2^ cfu/mL: 1.90-fold, IQR: 1.47–3.19, *p* < 0.05; 10^4^ cfu/mL: 2.47-fold, IQR: 1.60–3.18, *p* < 0.05, Figure 2G), while the probiotic mix did not affect the mRNA expression of CXCL1, CXCL2, CXCL6 and CCL5.

We also continued to measure the protein expression of CXCL1, CXCL8, CXCL10 and CCL2 after the stimulation with the probiotic mix or with each probiotic strain alone. We chose to measure the protein expression of these specific chemokines for two reasons; first, these chemokines are known to have a significant role in intestinal inflammation, and secondly, in our study, the mRNA expression of these chemokines was mostly affected by the probiotics.

Regarding the protein levels of CXCL1, the higher concentration resulted in a mild but not statistically significant upregulation (Figure 2I), while neither concentration significantly altered the protein secretion of CXCL8 (Figure 2J) and CCL2 (Figure 2K). CXCL10 was found to be undetectable in all samples (data not shown).

**Figure 3:**
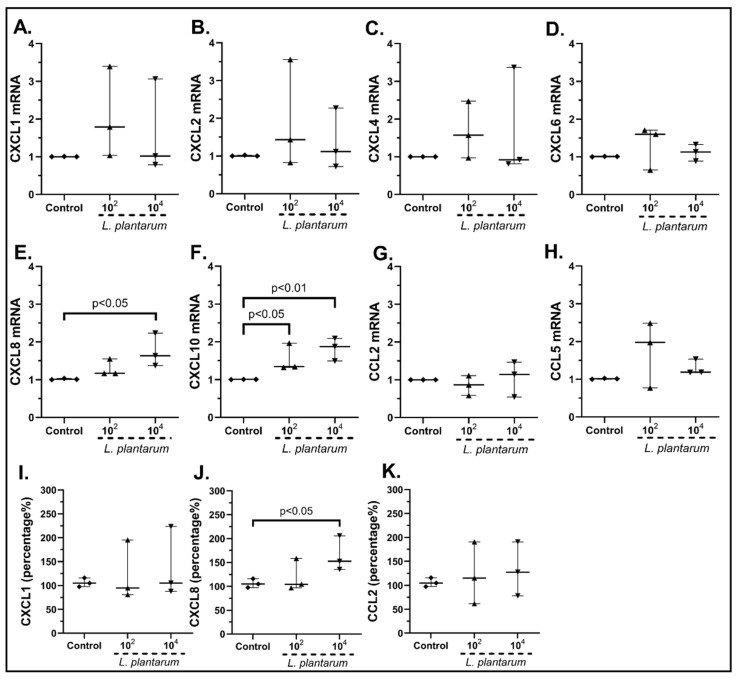
cSEMFs mRNA expression of CXCL1 (**A**), CXCL2 (**B**), CXCL4 (**C**), CXCL6 (**D**), CXCL8 (**E**), CXCL10 (**F**), CCL2 (**G**) and CCL5 (**H**) and protein levels of CXCL1 (**I**), CXCL8 (**J**) and CCL2 (**K**) after stimulation with *Lactiplantibacillus plantarum.* Results are presented as median with interquartile range. N = 3.

Having observed that the probiotic mix has a diverse outcome on SEMFs’ chemokine production, we proceeded to examine the effect of each probiotic strain alone. The stimulation of cSEMFs with 10^2^ cfu/mL and 10^4^ cfu/mL *Lactiplantibacillus plantarum* resulted in the increased mRNA expression of CXCL10 (10^2^ cfu/mL: 1.44-fold, IQR: 1.32–1.96, *p* < 0.05, 10^4^ cfu/mL: 1.87-fold, IQR: 1.49–2.09, *p* < 0.01, Figure 3F), while its lower dose also induced the mRNA of CXCL8 (1.63-fold, IQR: 1.38–2.23, *p* < 0.05, Figure 3E). In addition, no statistically significant effect was observed on the mRNA production of CXCL1 (Figure 3A), CXCL2 (Figure 3B), CXCL4 (Figure 3C), CXCL6 (Figure 3D), CCL2 (Figure 3G) and CCL5 (Figure 3H).

Regarding the protein levels, although *Lactiplantibacillus plantarum* had no effect on CXCL1 (Figure 3I) and CCL2 (Figure 3K) protein production, its higher dose did indeed induce the protein expression of CXCL8 (152.3%, IQR: 135.6–205.8%, *p* < 0.05, Figure 3J). Again, CXCL10 was found undetectable in all samples (data not shown).

**Figure 4:**
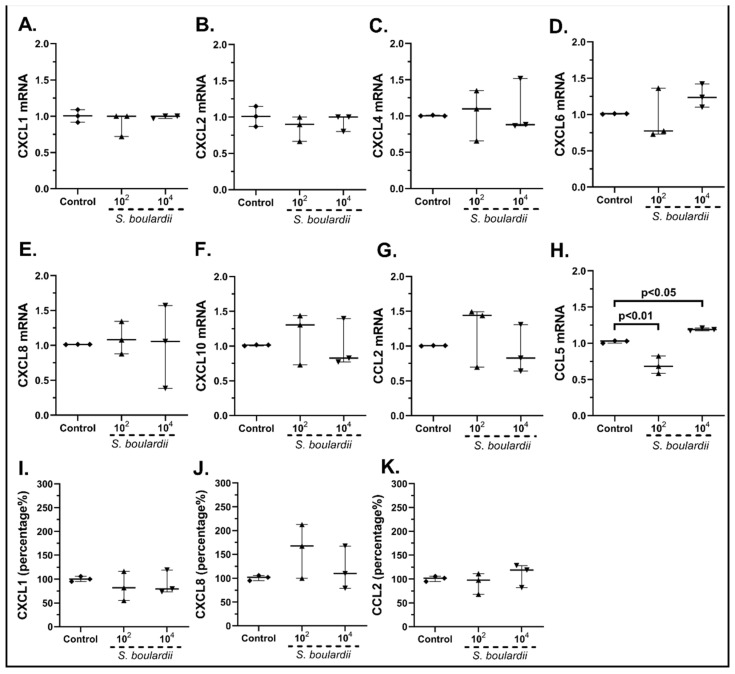
cSEMF mRNA expression of CXCL1 (**A**), CXCL2 (**B**), CXCL4 (**C**), CXCL6 (**D**), CXCL8 (**E**), CXCL10 (**F**), CCL2 (**G**) and CCL5 (**H**) and protein levels of CXCL1 (**I**), CXCL8 (**J**) and CCL2 (**K**) after stimulation with *Saccharomyces boulardii.* Results are presented as median with interquartile range. N = 3.

*Saccharomyces boulardii* also affected, in a lesser way, the expression of cSEMFs, with 10^2^ cfu/mL downregulating CCL5 mRNA (0.68-fold, IQR: 0.58–0.82, *p* < 0.01, Figure 4H) and with 10^4^ cfu/mL upregulating it (1.89-fold, IQR: 1.17–1.21, *p* < 0.05, Figure 4H). As no effect on the mRNA expression of the rest of the studied chemokines (Figure 4A–G) was seen, we proceeded to examine whether *Saccharomyces boulardii* could affect the expression of any other chemokine, finding that the 10^4^ cfu/mL could increase the mRNA expression of CXCL5 (1.37-fold, IQR: 1.30–1.46, *p* < 0.01, Supplementary Figure S1).

As for protein chemokine production, although *Saccharomyces boulardii* did not affect the expression of CXCL1 (Figure 4I) and CCL2 (Figure 4K), it slightly, but not statistically significantly, upregulated the expression of CXCL8 (Figure 4J). CXCL10 was also found undetectable in our samples (data not shown).

**Figure 5:**
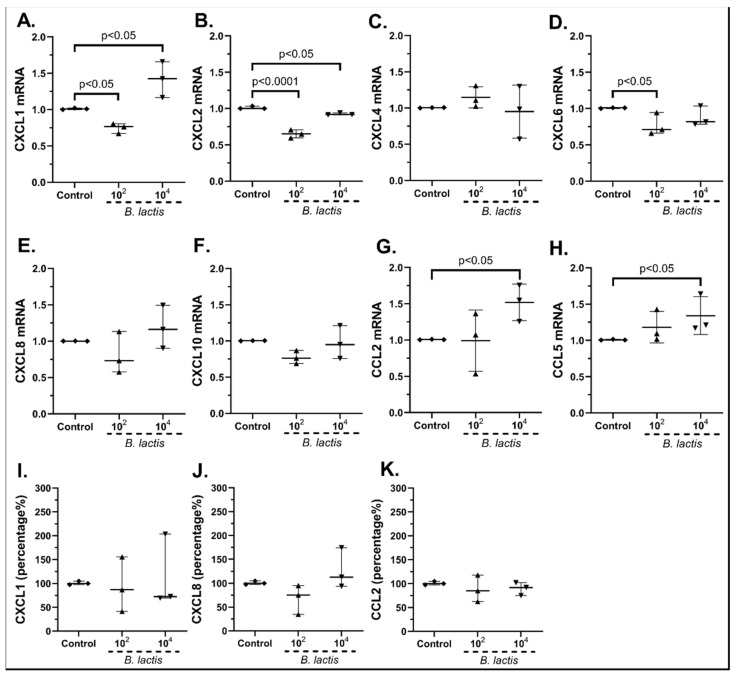
cSEMF mRNA expression of CXCL1 (**A**), CXCL2 (**B**), CXCL4 (**C**), CXCL6 (**D**), CXCL8 (**E**), CXCL10 (**F**), CCL2 (**G**) and CCL5 (**H**) and protein levels of CXCL1 (**I**), CXCL8 (**J**) and CCL2 (**K**) after stimulation with *Bifidobacterium lactis.* Results are presented as median with interquartile range. N = 3.

Regarding *Bifidobacterium lactis*, the 10^2^ cfu/mL reduced the mRNA production of CXCL1 (0.77-fold, IQR: 0.67–0.80, *p* < 0.05), although the 10^4^ cfu/mL increased it (1.42-fold, IQR: 1.16–1.66, *p* < 0.05, Figure 5A). Furthermore, 10^2^ cfu/mL of *B. lactis* downregulated CXCL6 mRNA (0.71-fold, IQR: 0.66–0.94, *p* < 0.05, Figure 5D), while 10^4^ cfu/mL increased CCL2 (1.55-fold, IQR: 1.26–1.75, *p* < 0.05, Figure 5G) and CCL5 mRNA expression (1.21-fold, IQR: 1.17–1.64, *p* < 0.05, Figure 5H), and both concentrations downregulated the mRNA expression of CXCL2 (10^2^ cfu/mL: 0.65-fold, IQR: 0.59–0.70, *p* < 0.0001, 10^4^ cfu/mL: 0.92-fold, IQR: 0.91–0.94, *p* < 0.05, Figure 5B). No effect was observed on the mRNA expression of CXCL4 (Figure 5C), CXCL8 (Figure 5E) or CXCL10 (Figure 5F).

The protein levels of CXCL1 (Figure 5I), CXCL8 (Figure 5J) and CCL2 (Figure 5K) were not statistically significantly affected by the probiotic strain, but were observed to have the same tendency compared with their mRNA levels. Once again, CXCL10 was found undetectable (data not shown).

**Figure 6:**
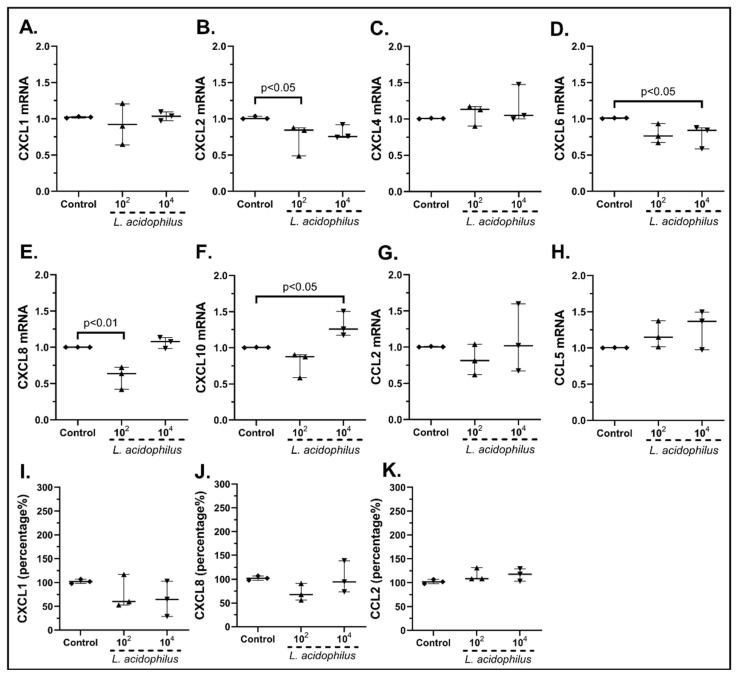
cSEMF mRNA expression of CXCL1 (**A**), CXCL2 (**B**), CXCL4 (**C**), CXCL6 (**D**), CXCL8 (**E**), CXCL10 (**F**), CCL2 (**G**) and CCL5 (**H**) and protein levels of CXCL1 (**I**), CXCL8 (**J**) and CCL2 (**K**) after stimulation with *Lactobacillus acidophilus.* Results are presented as median with interquartile range. N = 3.

Last but not least, the *Lactobacillus acidophilus* stimulation of cSEMFs also decreased the mRNA expression of chemokines such as CΧCL2 (10^2^ cfu/mL: 0.84-fold, IQR: 0.49–0.87, *p* < 0.05, Figure 6B), CXCL6 (10^4^ cfu/mL: 0.84-fold, IQR: 0.58–0.87, *p* < 0.05, Figure 6D) and CXCL8 (10^2^ cfu/mL: 0.63-fold, IQR: 0.42–0.72, *p* < 0.01, Figure 6E). Regarding the rest of the studied chemokines, *L. acidophilus* stimulation led to a statistically significant upregulation of the CXCL10 mRNA (10^4^ cfu/mL: 1.26-fold, IQR: 1.17–1.50, *p* < 0.05, Figure 6F) and had no statistically significant impact on the mRNAs of CXCL1 (Figure 6A), CXCL4 (Figure 6C), CCL2 (Figure 6G) and CCL5 (Figure 6H).

The protein secretion of CXCL8 was also decreased after the 10^2^ cfu/mL stimulation, although it did not reach statistical significance (Figure 6J), while neither concentration of *L. acidophilus* altered the protein levels of CXCL1 (Figure 6I) and CCL2 (Figure 6K). CXCL10 was found undetectable (data not shown).

Apart from the aforementioned chemokines, we also investigated the effect of probiotic strains of the mRNA expression of CXCL3, CXCL5, CXCL11, CXCL12 and CXCL14, but their effects were not statistically significant and, therefore, are presented in Supplementary Figure S1. As far as CXCL7, CXCL9 and CCL20 mRNAs are concerned, they were not expressed neither by unstimulated cSEMFs nor after the probiotics were added (data not shown).

#### 2.3.2. The Probiotic Strains Regulate the Expression of Wound-Healing-Related Factors on cSEMFs

As we observed that most of the probiotic strains either induce a mild upregulation or downregulation of various chemokines associated with inflammation, we next investigated their effect on the wound healing process through the expression of various wound-healing-related factors and the migration capacity of cSEMFs. Unstimulated cSEMFs had a baseline mRNA expression of Collagen type I, type III, Fibronectin, α-SMA and Tissue Factor, as well as an average collagen secretion and migration rate.

**Figure 7:**
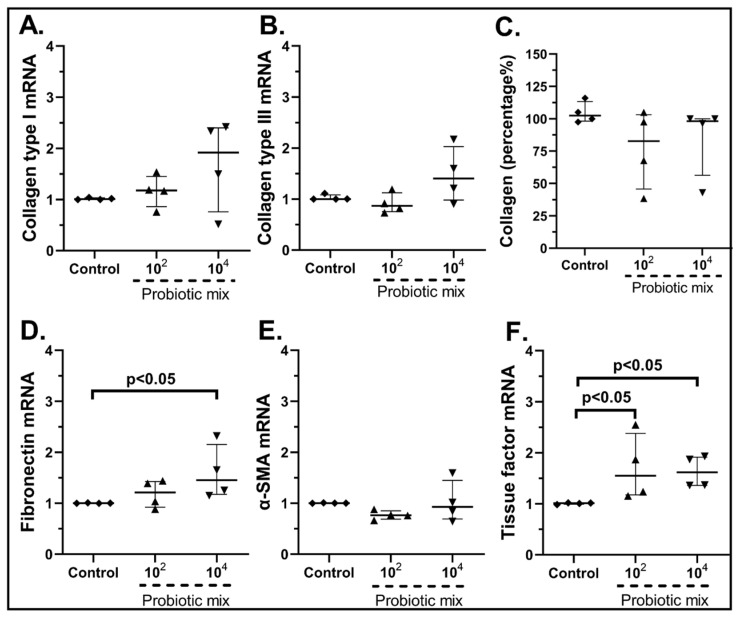
cSEMF mRNA expression of Collagen Type I (**A**), Collagen Type III (**B**), Fibronectin (**D**), α-SMA (**E**), Tissue factor (**F**) and Collagen secretion (**C**) after stimulation with the probiotic mix of *Bifidobacterium lactis, Lactobacillus acidophilus, Lactiplantibacillus plantarum* and *Saccharomyces boulardii*. Results are presented as median with interquartile range. N = 4.

Regarding collagen production, the probiotic mix had no statistically significant effect on mRNA Collagen Type I (Figure 7A), Collagen Type III (Figure 7B) or its total protein production (Figure 7C) or α-SMA (Figure 7F). Fibronectin was found upregulated when cSEMFs were stimulated with the higher probiotic dose (1.45-fold, IQR: 1.17–2.15, *p* < 0.05, Figure 7D), while both concentrations augmented the mRNA of Tissue Factor (10^2^ cfu/mL: 1.15-fold, IQR: 1.17–2.38, *p* < 0.05; 10^4^ cfu/mL: 1.61-fold, IQR: 1.36–1.92, *p* < 0.05, Figure 7F).

**Figure 8:**
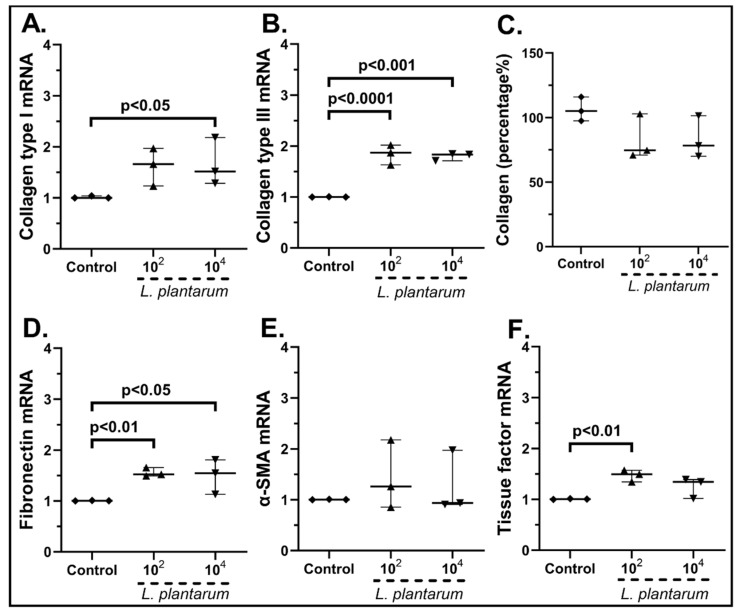
cSEMF mRNA expression of Collagen Type I (**A**), Collagen Type III (**B**), Fibronectin (**D**), α-SMA (**E**), Tissue factor (**F**) and Collagen secretion (**C**) after stimulation with *Lactiplantibacillus plantarum.* Results are presented as median with interquartile range. N = 3.

As far as the stimulation of cSEMFs with *Lactiplantibacillus plantarum* is concerned (Figure 8), both 10^2^ cfu/mL and 10^4^ cfu/mL increased the mRNA expression of Collagen Type III (10^2^ cfu/mL: 1.87-fold, IQR: 1.63–2.01, *p* < 0.0001; 10^4^ cfu/mL: 1.83-fold, IQR: 1.71–1.84, *p* < 0.0001, Figure 8B) and fibronectin (10^2^ cfu/mL, 1.52-fold, IQR: 1.49–1.65, *p* < 0.01; 10^4^ cfu/mL 1.54-fold, IQR: 1.13–1.80, *p* < 0.05 Figure 8D). Similarly, the higher probiotic dose resulted in upregulation of Collagen Type I (1.51-fold, IQR: 1.28–2.18, *p* < 0.05, Figure 8A), while the lower dose led to a statistically significant increase in Tissue Factor (1.49-fold, IQR: 1.34–1.57, *p* < 0.01 Figure 8F). Regarding α-SMA mRNA (Figure 8E) and total protein production (Figure 8C), this probiotic strain had no effect on their expression.

**Figure 9:**
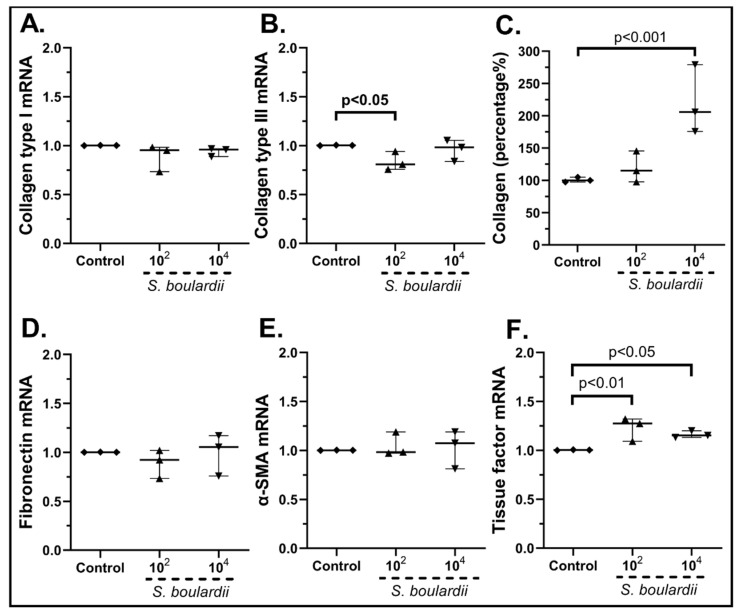
cSEMF mRNA expression of Collagen Type I (**A**), Collagen Type III (**B**), Fibronectin (**D**), α-SMA (**E**), Tissue factor (**F**) and Collagen secretion (**C**) after stimulation with *Saccharomyces boulardii.* Results are presented as median with interquartile range. N = 3.

When cSEMFs were stimulated with *Saccharomyces boulardii* (Figure 9), the impact was dependent on the concentration of the probiotic. On the one hand, 10^2^ cfu/mL of *S. boulardii* downregulated the mRNAs of Type ΙΙΙ (0.81-fold, IQR: 0.75–0.94, *p* < 0.05, Figure 9B), but on the other hand its highest dose resulted in a statistically significant increase in its total protein production (205.9%, IQR: 175.7–279.0%, *p* < 0.001, Figure 9C). In addition, both doses led to the upregulation of Tissue Factor (10^2^ cfu/mL: 1.27-fold, IQR: 1.09–1.32, *p* < 0.01; 10^4^ cfu/mL: 1.15-fold, IQR: 1.13–1.20, *p* < 0.05, Figure 9F), but neither dose had any effect on the mRNA expression of Collagen Type I (Figure 9A), Fibronectin (Figure 9D) and α-SMA (Figure 9E).

**Figure 10:**
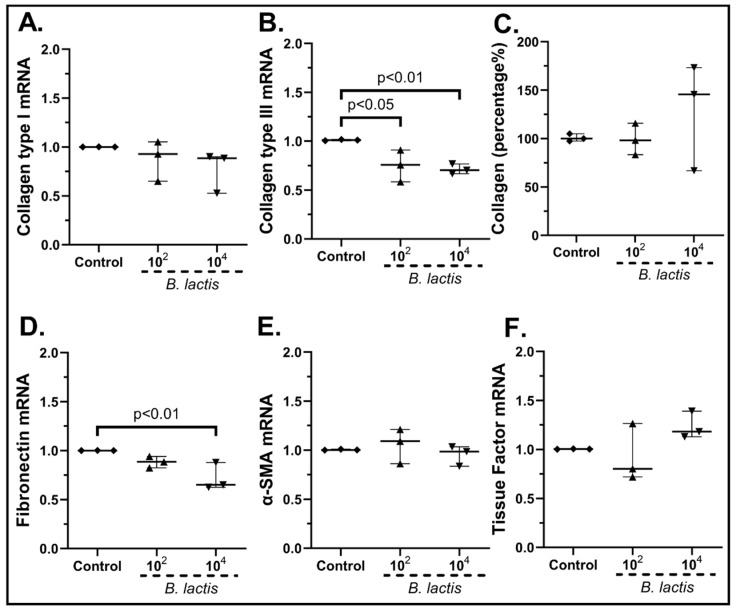
cSEMF mRNA expression of Collagen Type I (**A**), Collagen Type III (**B**), Fibronectin (**D**), α-SMA (**E**), Tissue factor (**F**) and Collagen secretion (**C**) after stimulation with *Bifidobacterium lactis.* Results are presented as median with interquartile range. N = 3.

*Bifidobacterium lactis* added alone (Figure 10) reduced the cSEMF mRNA of Collagen Type ΙΙΙ (10^2^ cfu/mL: 0.76-fold, IQR: 0.58–0.90, *p* < 0.05; 10^4^ cfu/mL: 0.70-fold, IQR: 0.67–0.76, *p* < 0.01, Figure 10B), but had no effect on Collagen Type I mRNA (Figure 10A) or its total protein production (Figure 10C). Similarly, the addition of the highest dose resulted in the downregulation of Fibronectin (10^4^ cfu/mL: 0.65-fold, IQR: 0.62–0.88, *p* < 0.01, Figure 10D), while none of the two doses had any effect on α-SMA (Figure 10E) and Tissue Factor (Figure 10F) mRNA expression.

**Figure 11:**
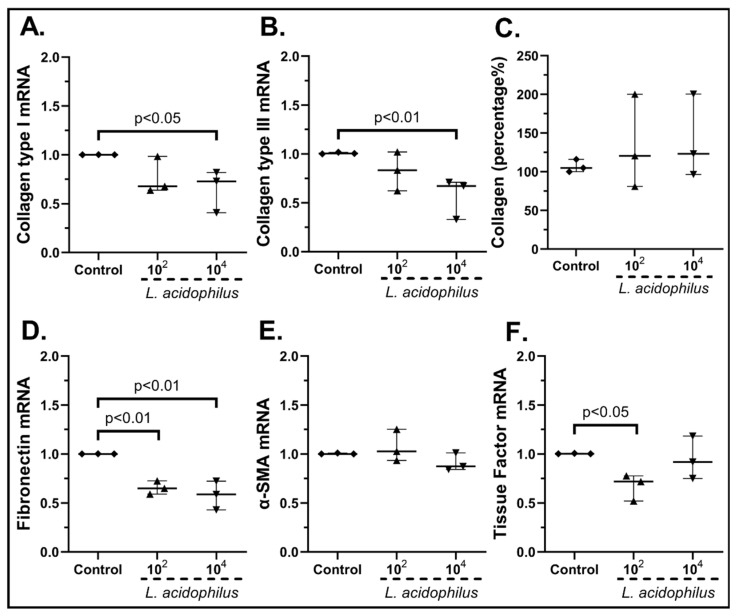
cSEMF mRNA expression of Collagen Type I (**A**), Collagen Type III (**B**), Fibronectin (**D**), α-SMA (**E**), Tissue factor (**F**) and Collagen secretion (**C**) after stimulation with *Lactobacillus acidophilus.* Results are presented as median with interquartile range. N = 3.

*Lactobacillus acidophilus* stimulation of cSEMFs (Figure 11) decreased the mRNA expression of Collagen Type I (10^4^ cfu/mL: 0.73-fold, IQR: 0.41–0.82, *p* < 0.05, Figure 11A) and Type ΙΙΙ (10^4^ cfu/mL: 0.67-fold, IQR: 0.33–0.71, *p* < 0.01, Figure 11B), but had no effect on its total protein production (Figure 11C). Fibronectin and Tissue Factor were found downregulated when cSEMFs were stimulated either with both doses (10^2^ cfu/mL: 0.65-fold, IQR: 0.59–0.73, *p* < 0.01; 10^4^ cfu/mL: 0.59-fold, IQR: 0.43–0.72, *p* < 0.01, Figure 11D) or with the lower dose (10^2^ cfu/mL: 0.72-fold, IQR: 0.52–0.78, *p* < 0.05, Figure 11F), respectively. No effect on α-SMA mRNA expression was seen (Figure 11E).

#### 2.3.3. The Probiotic Strains Promote Wound Healing through the Induction of cSEMFs’ Migration

**Figure 12:**
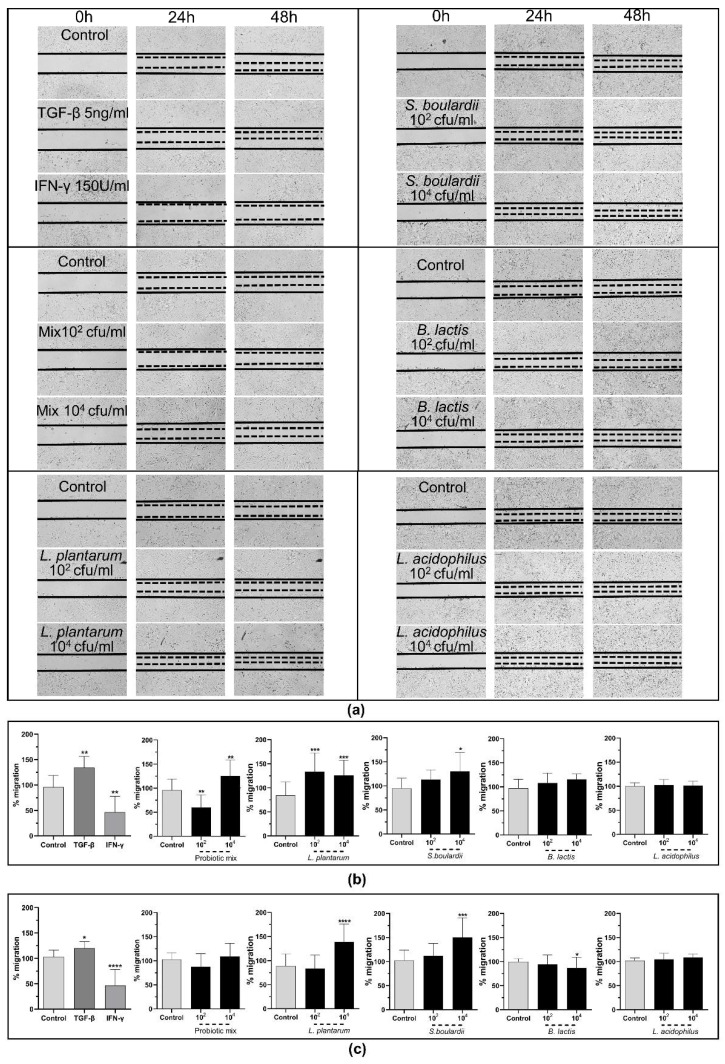
(**a**) cSEMF migration after stimulation with the probiotic mix of *Bifidobacterium lactis, Lactobacillus acidophilus, Lactiplantibacillus plantarum* and *Saccharomyces boulardii* and each probiotic strain alone after 24 h and 48 h and percentage of the migratory rate (**b**) after 24 h and (**c**) after 48 h. Statistical significance is marked as a *p*-value compared to unstimulated cSEMF migration (control). * *p* < 0.05; ** *p* < 0.01; *** *p* < 0.001; **** *p* < 0.0001. Results are presented as median with interquartile range. N = 3.

We next proceeded to study the effect of these probiotics on SEMFs’ migration rate. As it is already known, TGF-β positively affects the migration rate of cSEMFs, while IFN-γ acts negatively [51], and thus, we used these two stimulators as positive and negative controls, respectively. As seen in Figure 12, TGF-β increased the migration rate (24 h: 129.5%, IQR: 115.7–151.77%, *p* < 0.01; 48 h: 124.1%, IQR: 104.3–131%, *p* < 0.05), while IFN-γ inhibited it (24 h: 43.97%, IQR: 16.85–79.50%, *p* < 0.01; 48 h: 43.97%, IQR: 16.85–79.50%, *p* < 0.0001).

Regarding the effect of the probiotic strains, the cSEMFs’ migration rate was reduced by 41.76% (IQR: 34.8–79.8%) when stimulated with 10^2^ cfu/mL of the probiotic mix for 24 h (*p* < 0.01, Figure 12b), in contrast to stimulation with 10^4^ cfu/mL of the mix that promoted it by 133.8% (IQR: 102.8–150.2%) after 24 h (*p* < 0.01, Figure 12b). No effect was observed at 48 h of stimulation (Figure 12c), suggesting that the probiotic mix probably affects the migration rate during the early phase of wound healing.

When each probiotic strain alone was used, both 10^2^ cfu/mL and 10^4^ cfu/mL of *Lactiplantibacillus plantarum* increased the cSEMF migratory rate after 24 h (10^2^ cfu/mL: 128.3%, IQR: 100.6–161.8%, *p* < 0.05; 10^4^ cfu/mL: 124.1%, IQR: 96.1–156.1%) *p* < 0.05, Figure 12b) and the increase caused by 10^4^ cfu/mL continued after 48 h as well (by 146.9%, IQR: 101.8–175.2%, *p* < 0.0001, Figure 12c). The 10^4^ cfu/mL of *Saccharomyces boulardii* promoted the migration of cSEMFs by 130.2% after 24 h (IQR: 82.1–169.3%, *p* < 0.05, Figure 12b) and by 150.3% after 48 h (IQR: 107.1–191.2%, *p* < 0.001, Figure 12c) too. Although the stimulation of cSEMFs with *Bifidobacterium lactis* had no effect at 24 h (Figure 12b), it reduced their migratory rate after 48 h (10^4^ cfu/mL: 86.9%, IQR: 64.0–109.2%, *p* < 0.05, Figure 12c) and *Lactobacillus acidophilus* stimulation did not affect it significantly (Figure 12b,c).

## 3. Discussion

In this study, we showed that the combination of *Bifidobacterium lactis, Lactobacillus acidophillus, Lactiplantibacillus plantarum* and *Saccharomyces boulardii* induces a mild inflammation by elevating the mRNA of specific chemokines while also promoting wound healing in colonic subepithelial myofibroblasts, as they upregulate both the expression of wound-healing-related factors and their migration rate. We also showed that each probiotic strain has a different effect on cSEMFs’ inflammatory and healing behavior, with some strains upregulating or downregulating the expression of chemokines and healing factors, and therefore, combining these strains results in a more balanced and favorable situation for mucosal immunity and wound healing. In addition, we also showed that, even though almost all of the probiotic strains did not greatly induce the protein expression of CXCL1, CXCL8, CXCL10 and CCL2, they did have a mild effect on their protein production, which did not reach statistical significance. This result suggests that this mild inflammatory response in cSEMFs could be interpreted, not as a pathological inflammatory response, but rather as a possible immune alertness, contributing in this way to the host’s defenses.

We should also underline that the reason for measuring the protein expression of these specific chemokines lies on two factors. First, it is already known that these chemokines have a significant role in intestinal inflammation, and secondly, in our study, the mRNA expression of these chemokines was mostly affected by the probiotics. Nonetheless, we do acknowledge that the fact of not investigating the protein expression of all the studied chemokines may be a limitation in our study.

Importantly, it should be noted that the probiotics remained viable in the cSEMF culture medium, proving that not only do the bacteria and fungi affect the human cells, but the metabolome of both the mucosa and the microflora can react to changes and inflammatory signals in the microenvironment.

To our knowledge, this is the first study that attempts to identify the effect of this probiotic mix, as well as of each strain alone, on primary human cSEMFs. Normally, cSEMFs lie beneath the epithelium [47], so this direct contact with the live probiotics, as displayed in the current study, and not just their secretome, can be more beneficial [57] due to simulating an ulcer or a trauma in need of the activation of cSEMFs for the initiation of the healing process. It is also worth mentioning that, when studying the effects of probiotics, mostly cell lines and animal models are used. The use of primary human cells in the current study provides more individualized and less normalized results without the need to endanger patients, while the recruitment of human colonoids and human intestinal organoids [58], which consist of both the mesenchymal and epithelial structures of the gut [48], could further enrich these results.

In particular, *L. plantarum* induced the mRNA and protein expression of CXCL1 (Figure 3A,I) and CXCL8 (Figure 3E,J) as well as the mRNA expression of CXCL10 (Figure 3F) and CCL5 in human cSEMFs (Figure 3H), agreeing with CXCL1 induction in mice [59] and the lack of effect on CCL2 production in Caco-2 cells [60]. However, the suppression of CXCL1, CXCL10 and CCL2 observed in C57BL/6 and *MyD88^−/−^* mice [61] did not correlate with the results of the current study. Regarding the *S. boulardii* stimulation of cSEMFs, it resulted in both the upregulation and downregulation of CCL5 mRNA, based on the concentration used (Figure 4D), with no previous research on its impact on those chemokines. The higher dose of the probiotic strain *B. lactis* upregulated CCL2 and CCL5 mRNAs (Figure 5G,H, respectively), despite downregulating them on BALF of Schizophrenic patients when co-administered with *Lactobacillus rhamnosus* [62] and on TNBS-induced colitis mice when co-administered with *Lactobacillus plantarum* and *Streptococcus thermophilus* [63]. *B. lactis* alone also downregulated cSEMFs CXCL6 mRNA expression (Figure 5D) and had a different effect on the mRNAs of CXCL1 (Figure 5A) and CXCL2 (Figure 5B), depending on its concentration. Last but not least, *L. acidophilus* resulted in the downregulated CXCL2, CXCL6 and CXCL8 mRNAs (Figure 6B,D,E) as well as on the slight downregulation of CXCL1 and CXCL8 protein levels (Figure 6I,J), in accordance with the reduction in CXCL2 in 5-fluorouracil-induced intestinal mucositis in mice [64] and the reduction in CXCL8, along with CCL2, in bovine mammary epithelial cells [65].

Overall, we showed that each probiotic strain alone could induce or ameliorate a variety of chemokines, mainly focusing on CXCL1, CXCL2, CXCL4, CXCL6, CXCL8, CXCL10, CCL2 and CCL5, which affect different immunological Th1, Th2, Th17 and/or Treg downward cascades. The observed immunological stimulation by the single strains is mild and can simulate the dysbiotic conditions in the gut mucosa, since either higher or lower concentrations of any strain of the microflora could be both the reason and the consequence of a pathological condition [2]. In contrast, when all of the four probiotics were used as a stimulus on cSEMFs, the effect was more balanced, as almost all studied chemokines were mildly upregulated but did not cause an exacerbated inflammation. In addition, the upregulation of those chemokines can help to alert the gut mucosa of any intrusion or pathological condition and initiate attracting neutrophils (CXCL1, CXCL2, CXCL6 and CXCL8), natural killer cells (CXCL10 and CCL5) and macrophages (CXCL6, CXCL8, CCL2 and CCL5), among other innate immunity cells [66].

The advances of using combinations of multiple probiotic organisms to balance pro- and anti-inflammatory signals are highlighted, as described by other research teams too [67,68,69,70]. Indeed, we also observed that combining *B. lactis, L. acidophilus, L. plantarum* and *S. boulardii* resulted in a moderate, but statistically significant, upregulation of both chemokine and healing factor expression, highlighting again the importance of using multiple probiotics. This specific probiotic mix is already proven to reduce small intestinal bacterial overgrowth during irritable bowel syndrome [71] as well as to reduce infection incidents during surgeries [72,73], and we showed that it can produce stronger signals than the individual strains, not by fold change but by combining more chemokines while using the same concentrations. It should be noted that the effect of the mix is more similar to the effect of *L. plantarum* alone, despite being the less abundant microorganism in the mix.

Although *B. lactis* is known to promote PBMCs’ TGF-β production [70], our results support an opposite effect on SEMFs, by reducing not only their migratory rate after the 48 h stimulation (Figure 12c) but also the mRNA expression of Type III and Fibronectin (Figure 10B,D, respectively). This downregulation of Collagen Type I and Type III mRNAs by *B. lactis* (Figure 10B) and *L. acidophilus* (Figure 11A,B) could be correlated with the downregulated CXCL6 (Figure 5D and Figure 6D) [74], providing not only an anti-inflammatory but also an anti-fibrotic role for these bacteria. The mRNAs of the wound healing factors were also reduced by *S. boulardii* (Figure 9)*,* while the same effect is not apparent in the probiotic mix stimulation (Figure 7), probably due to the opposite effect of *L. plantarum* (Figure 8) once again. However, the secretion of total collagen was induced only by stimulating cSEMFs with *S. boulardii* alone (Figure 9C), hinting at the urge to investigate the impact of this fungus on the other collagen types [75] in order to better understand its effect and to avoid pro-fibrotic responses.

In general, probiotics are widely used due to their ability to improve wound healing conditions in a wide variety of models. For instance, it was shown that probiotic supplementation with either *Lactobacillus paracasei, B. lactis, Lactobacillus rhamnosus, L. acidophilus*, or with *Lactobacillus rhamnosus*, *Bifidobacterium longum* can accelerate trauma healing after surgeries, with better results when simultaneously using multiple strains [76,77,78]. The re-epithelialization in DSS-induced mice was also faster after treatment with *Bifidobacterium bifidum*, *L. acidophilus* and *Bacillus amyloliquefaciens* [79] In a study by Tsai et al., the co-administration of heat-killed *L. plantarum* and *Lactobacillus paracasei* promoted healing in mice [80], while Kazemi et al. showed that the extracts of *L. plantarum* and *Lactobacillus casei* promoted the proliferation of mesenchymal stem cells [81]. Furthermore, a four-strain supplement containing *L. acidophilus, L. plantarum, Lactobacillus rhamnosus* and *Enterococcus faecium* benefited epithelial tight junction integrity and promoted wound healing [82], and another one containing *L. acidophilus, L. plantarum, B. lactis* and *Bifidobacterium breve* can strengthen the epithelial barrier of the gut [83].

cSEMF migratory rate and the expression of wound-healing factors simulate the conditions of wound healing and scarring in the gut mucosa [47]. The fact that *L. plantarum* and *S. boulardii* can promote healing is not news, as previously discussed; however, it is important to search the different impacts caused by these probiotic strains both within different tissues and within different strains and combinations. Since *L. acidophillus* alone had no significant effect and *B. lactis* was able to reduce cSEMF migration, these strains could be combined with strains such as *L. plantarum* and *S. boulardii* that promoted it (Figure 12) and achieve a quicker migration, when the correct concentrations are used. The superior effect of *L. plantarum* on cSEMF migration than of *L. acidophilus* contradicts their effect on wound healing on Wistar rats [84]. In summary, our results highlight once again the importance of diversity and abundance of the microflora in supporting wound healing via regulating both the expression of healing factors and the migration of cSEMFs to the trauma region.

This study highlights that combining the probiotic properties of a variety of organisms can better alert the gut immune system during pathological conditions than the use of single probiotic strains. Probiotic supplements should be further evaluated as an auxiliary treatment option for inflammatory diseases, since restoring the microflora interactions could contribute towards achieving symbiosis. As long as the pathways leading to fibrosis are taken into consideration, the healing effect can be supported by the administration of the correct combinations and concentrations of probiotic strains.

## 4. Materials and Methods

### 4.1. Patients

Colonic tissue was obtained endoscopically from healthy individuals, without systematic autoimmune disease or malignancy, who underwent screening colonoscopy and had no pathological findings. The endoscopies were performed at the Endoscopy Department, University Hospital of Alexandroupolis, Greece. The local Research Ethics Committee approved this study, and patients provided their informed written consent before participation (Protocol number 14127/07-04-2021).

### 4.2. Colonic Subepithelial Myofibroblast Isolation and Culture

Colonic subepithelial myofibroblasts were isolated from colonic biopsies of healthy individuals as previously described [50]. Briefly, the biopsies were obtained in ice cold Hank’s Balanced Salt Solution (HBSS; Biosera, Nuaille, France) with Ca^2+^/Mg^2+^, containing penicillin (P;100 U/mL; Biosera, Nuaille, France), streptomycin (S; 100 μg/mL; Biosera, Nuaille, France), amphotericin B (A; 2.5 μg/mL; Biosera, Nuaille, France) and gentamycin (G; 50 μg/mL; Biosera, Nuaille, France). After some short washes in HBSS with and without Ca^2+^/Mg^2+^, the biopsies were de-epithelialized for 15 min in 1 mM dithiothreitol (DTT, Sigma-Aldrich, Darmstadt, Germany), followed by 3 half-hour incubations with 1 mM Ethylene-Diamine tetraacetic Acid (EDTA, Sigma-Aldrich, Darmstadt, Germany) at 37 °C. The tissues were then placed in 75 cm^2^ flasks containing RPMI 1640 (PAN Biotech, Aidenbach, Germany) supplemented with 10% *v/v* Fetal Bovine Serum (FBS; Biosera, Nuaille, France) and the aforementioned antibiotics and kept in 5% CO_2_ at 37 °C for 4 weeks, with the medium being changed every day for 4 days and then twice a week. Once numerous myofibroblast colonies started to form, the biopsies were removed and the cells were cultured in Dulbecco’s Modified Eagle Medium (DMEM, 4.5 g/L glucose; PAN Biotech, Aidenbach, Germany) plus 10% FBS and P/S/A in 5% CO_2_ at 37 °C. The myofibroblast phenotype was verified with immunofluorescence microscopy as being α-smooth muscle actin (α-SMA) and vimentin positive and desmin negative using a fluorescent microscope (Leica DM2000, Leica Microsystems GmbH, Wetzlar, Germany) (Supplementary Figure S2). All experiments were performed with cSEMFs at passages 2–6 cultured in 6-well plates with FBS- and antibiotics-free DMEM until 95% confluence.

### 4.3. Probiotics

The probiotics *Bifidobacterium lactis* BB-12, *Lactobacillus acidophillus* LA-5, *Lactiplantibacillus plantarum* UBLP 40, *Saccharomyces boulardii* Unique-28 and their combined mixture were supplemented in lyophilized form and kindly provided by UNI-PHARMA S.A. Pharmaceutical Laboratories (Athens, Greece). They were reconstituted in FBS- and antibiotics-free DMEM (PAN Biotech, Aidenbach, Germany) and cultured in 5% CO2 at 37 °C for an hour prior to the cSEMF stimulation experiments.

### 4.4. Probiotic Viability Assay

The viability of the probiotic mix of *Bifidobacterium lactis* (1.75 BU/g), *Lactobacillus acidophillus* (1.75 BU/g)*, Lactiplantibacillus plantarum* (0.5 BU/g)*, Saccharomyces boulardii* (1.5 BU/g)*,* as well as of the strains alone was assessed using Trypan Blue (Gibco, Thermo Fisher Scientific Inc., Waltham, MA, USA). The lyophilized probiotics were reconstituted in FBS- and antibiotics-free DMEM (PAN Biotech, Aidenbach, Germany) and incubated in 5% CO_2_ at 37 °C for 48 h. Then, 10 μL of the probiotic solution was mixed with 10 μL Trypan Blue both at the beginning of the incubation (0 h) and, after 48 h, placed on a microscope slide and photographed at 40× and 100× using a light microscope (Leica DM2000, Leica Microsystems GmbH, Wetzlar, Germany).

### 4.5. Gram Staining

The probiotic mix and the strains *Bifidobacterium lactis, Lactobacillus acidophillus, Lactiplantibacillus plantarum* and *Saccharomyces boulardii* were stained using Gram staining (Sigma-Aldrich, Darmstadt, Germany) [85]. Specifically, the lyophilized strains were reconstituted in FBS- and antibiotics-free DMEM (PAN Biotech, Aidenbach, Germany) and 20 μL were heat-fixed on a glass slide. Then, Crystal Violet is used for 1 min, followed by Iodine for 1 min, followed by 1:1 acetone–ethanol mix for 5 s, and finally Safranin is used for 30′’ before the cells are seen using a light microscope (Leica DM2000, Leica Microsystems GmbH, Wetzlar, Germany).

### 4.6. cSEMF Probiotics Stimulation

The lyophilized mix of *B. lactis, L. acidophillus, L. plantarum* and *S. boulardii* and the strains alone were also reconstituted in FBS- and antibiotics-free DMEM. The cSEMFs were starved for 24 h and either left untreated or stimulated with 10^2^ or 10^4^ cfu/mL of the probiotic mix, or of the same concentration of each strain alone, (a) for 6 h, in order to study the mRNA expression of wound-healing-related factor and chemokines by quantitative PCR, and (b) for 48 h, when cSEMFs’ migration rate was examined via Wound Healing Assay and protein collagen via Sircol Assay.

### 4.7. RNA Extraction, cDNA Synthesis and Real-Time PCR

After 6 h incubation with the probiotics, cSEMFs were collected using 500 μL Nucleozol (MACHEREY-NAGEL, Düren, Germany) and RNA extraction was performed according to manufacturer’s instructions and as previously described [86]. In brief, 200 μL H_2_O were added to each tube, centrifuged and the upper phase was mixed with 500 μL Isopropanol, before being further centrifuged. The pellet was washed twice with 75% Ethanol and total RNA was measured using the Quawell Q5000 UV-Vis Spectometer (Quawell, San Jose, CA, USA). Any DNA contamination was removed using Deoxyribonuclease I (TaKaRa, Kusatsu, Shiga, Japan) that was later EDTA- and heat-inactivated. Then, 250 ng of RNA was reverse-transcribed into cDNA using the PrimeScript 1st strand cDNA Synthesis Kit (TaKaRa, Kusatsu, Shiga, Japan), according to the manufacturer’s instructions. Finally, the mRNA expression of the chemokines CCL2, CCL5, CCL20, CXCL1, CXCL2, CXCL3, CXCL4, CXCL5, CXCL6, CXCL7, CXCL8, CXCL9, CXCL10, CXCL11, CXCL12 and CXCL14 as well as collagen type I, III, fibronectin, Tissue Factor and α-SMA (Table 1) was assessed by quantitative Real-Time PCR using Sybr Green (Kapa Biosystems, Wilmington, CA, USA) in SaCycler-96 RUO (Sacace Biotechnologies, Como, Italy). A two-step amplification protocol was performed for all studied genes, except CXCL9, CXCL10 and TF, and the gene expression of each studied gene was normalized against GAPDH gene expression in the same sample using the 2^−ΔΔCt^ method [87].

### 4.8. Enzyme-Linked Immunosorbent Assay (ELISA)

cSEMF production of the chemokines CXCL1, CXCL8, CXCL10 and CCL2 was measured using the Human DuoSet^®^ ELISAs (R&D Systems, Minneapolis, MN, USA) kit according to manufacturer’s instructions as previously described [48]. In short, cSEMFs were cultured in 6-well plates until 95% confluence, starved for 24 h and then stimulated with 10^2^ or 10^4^ cfu/mL of the probiotics (mix or each strain alone) for 48 h, as already described, and their supernatants were collected. Then, 96-well plates were coated overnight with capture antibody for each chemokine and the next day were incubated with the recommended blocking buffer for 1.5 h. Duplicates of each sample as well as known concentrations of each chemokine were added and incubated for 2 h. Then, biotinylated detection antibody for each chemokine was added for another 2 h, followed by adding Streptavidin–horseradish peroxidase for 20 min and tetramethylbenzidine with H_2_O_2_ for another 20 min in order to produce different optical densities (OD) of color, which were measured at 450 nm on a microplate reader (Diareader EL × 800; Dialab, Wr. Neudorf, Austria). The chemokine concentration was calculated using a linear standard curve.

### 4.9. Wound Healing Assay after Probiotic Stimulation

cSEMFs were cultured in 6-well plates until 95% confluence and starved for 24 h. Then, mechanical trauma was caused and cSEMFs were stimulated with 10^2^ or 10^4^ cfu/mL of the probiotics (mix or each strain alone) for 48 h, as it has been already described above. Photographs of multiple regions of the trauma were taken at time 0 h and after 24 h and 48 h using an inverted cell culture microscope (Olympus CKX53 LED, OLYMPUS EUROPA SE & CO. KG Hamburg, Germany). The cSEMF migratory rate was estimated as the average percentage of the area closure. TGF-β1 (5 ng/mL) and IFN-γ (150 U/mL) were used as the positive and negative controls of migration, respectively, as it has been already shown in a previous publication from our research team [50].

### 4.10. Collagen Production after Probiotic Stimulation

cSEMFs’ collagen production was measured using the Sircol assay (Sircol; Biocolor, Carrickfergus, UK), according to manufacturer’s instructions and as previously described [86]. In summary, cells were incubated with 10^2^ or 10^4^ cfu/mL of the aforementioned probiotics (mix or each strain alone) for 48 h and their supernatants were concentrated overnight with Polyethylene glycol in Tris-HCl Buffer. Then, the samples were centrifuged at 12,000× *g* for 10 min and the supernatants were discarded before adding Sirius Red for 30 min. After the incubation, the mixtures were centrifuged at 12,000× *g* for 10 min, washed with ice cold Acid-Salt Wash, and the collagen pellet was dissolved in 0.5 M NaOH Alkali Reagent. The optical densities (ODs) of the samples and controls of known collagen concentration were measured at 540 nm in a microplate reader (Diareader EL × 800; Dialab, Wr. Neudorf, Austria). The collagen concentration was calculated using a linear standard curve.

### 4.11. Statistical Analysis

Statistical analyses were performed using Prism Software 9 (GraphPad Software, San Diego, CA, USA. Access date, 12 May 2020). The results of this study comprise three independent experiments per stimulation presented as medians with interquartile ranges (IQRs) and were analyzed using an ordinary one-way ANOVA with a follow-up Fisher’s LSD Test. Statistical significance was established at an alpha level *p* < 0.05.

## 5. Conclusions

The current study highlighted that the use of probiotics has a beneficial effect on gut mucosal immunity and healing. Our results underline that the use of the probiotic mixture has a better impact on regulating the chemokine expression than single strains, contributing to the alertness of the normal mucosal immune system and possibly to the regulation of the mechanisms that govern intestinal inflammation. Regarding mucosal healing, both the mixture and *L. plantarum* and *S. boulardii* alone had similar beneficial effects on cSEMFs, as they upregulated the production of various healing factors and promoted migration. Therefore, taking into consideration the effects on both immunity and healing, the use of the probiotic mix offers greater advantages. Further research is needed in order to identify the key interactions between the microflora and the human host, as well as to elucidate their implication both in symbiosis and the pathology of intestinal conditions.

## Figures and Tables

**Table 1:** Gene specific primer sequences and Tm used in this study.

Gene	Forward	Reverse	Tm (°C)
CCL2	AGGAAGATCTCAGTGCAGAGG	AGTCTTCGGAGTTTGGGTTTG	60
CCL5	CTCGCTGTCATCCTCATTGCT	TGTGGTGTCCGAGGAATATGG	60
CCL20	GCTGCTTTGATGTCAGTGC	GCAGTCAAAGTTGCTTGCTTC	56
Collagen Type I	CCCTGGAAAGAATGGAGATGAT	ACTGAAACCTCTGTGTCCCTTCA	60
Collagen Type III	GCTCTGCTTCATCCCACTATTA	TGCGAGTCCTCCTACTGCTAC	60
CXCL1	GCCCAAACCGAAGTCATAGCC	ATCCGCCAGCCTCTATCACA	60
CXCL2	GCTTGTCTCAACCCCGCATC	TGGATTTGCCATTTTTCAGCATCTT	60
CXCL3	CGCCCAAACCGAAGTCAT	GTGCTCCCCTTGTTCAGTATCT	60
CXCL4	GTCCAGTGGCACCCTCCTGA	AATTGACATTTAGGCAGCTGA	60
CXCL5	AGCTGCGTTGCGTTTGTTTAC	TGGCGAACACTTGCAGATTAC	60
CXCL6	AGAGCTGCGTTGCACTTGTT	GCAGTTTACCAATCGTTTTGGGG	60
CXCL7	TGAGACAGAATGAAACAC	AGGTGATGAATCTGCTG	60
CXCL8	TGGGTGCAGAGGGTTGTG	CAGACTAGGGTTGCCAGATTTA	60
CXCL9	AAGAAGCACGTGGTAAAACA	TCTCGGTGGCTATCTTGTTA	56
CXCL10	CCTGCTTCAAATATTTCCCT	CCTTCCTGTATGTGTTTGGA	56
CXCL11	GACGCTGTCTTTGCATAGGC	GGATTTAGGCATCGTTGTCCTTT	60
CXCL12	AGAGATGAAAGGGCAAAGAC	CGTATGCTATAAATGCAGGG	60
CXCL14	TCCGGTCAGCATGAGGCTCC	CACCCTATTCTTCGTAGACC	60
Fibronectin	CCAGTCCACAGCTATTCCTG	ACAACCACGGATGAGCTG	60
GapdH	GACATCAAGAAGGTGGTGAA	TGTCATACCAGGAAATGAGC	60
Tissue Factor	TTCAGTGTTCAAGCAGTGATTCC	ATGATGACCACAAATACCACAGC	51
α-SMA	AATGCAGAAGGAGATCACGG	TCCTGTTTGCTGATCCACATC	60

## Data Availability

Data is contained within the article and Supplementary Material.

## Supplementary Materials

The following supporting information can be downloaded at: https://www.mdpi.com/article/10.3390/ph15101293/s1, Figure S1: cSEMFs mRNA expression of CXCL3, CXCL5, CXCL11, CXCL12 and CXCL14 after stimulation with probiotics; Figure S2: Characterization of cSEMFs.
