# Supplementary figures and images for: The Probiotic Strains Bifidοbacterium lactis, Lactobacillus acidophilus, Lactiplantibacillus plantarum and Saccharomyces boulardii Regulate Wound Healing and Chemokine Responses in Human Intestinal Subepithelial Myofibroblasts
Source: Pharmaceuticals (Basel). 2022 Oct 20;15(10):1293. doi: 10.3390/ph15101293 (PMC9611312; doi:10.3390/ph15101293)

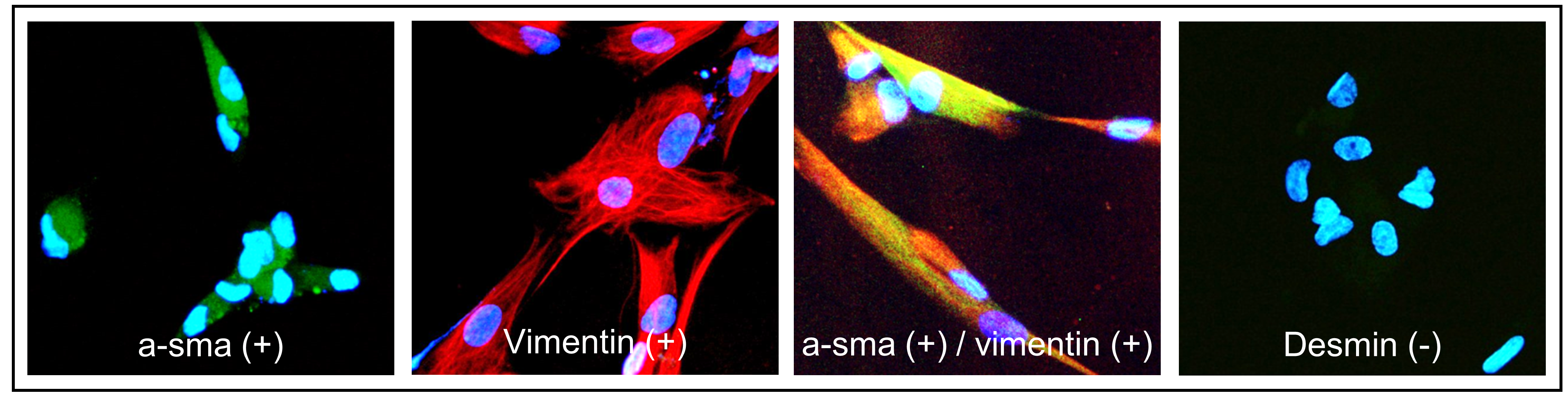

Supplement: Supplementary file 1 [file pharmaceuticals-15-01293-s001.zip › Supplementary Figure 2.tif]

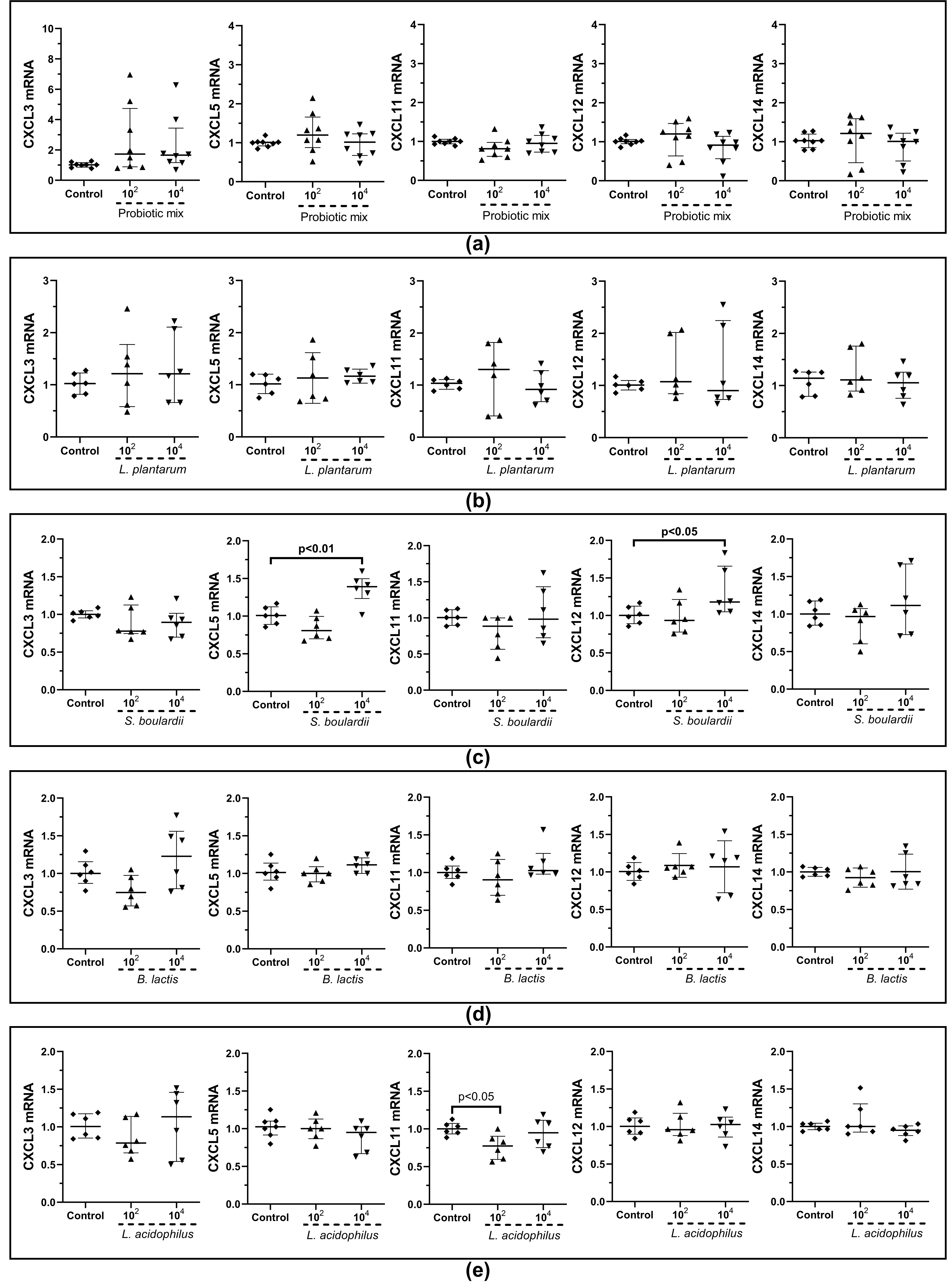

Supplement: Supplementary file 1 [file pharmaceuticals-15-01293-s001.zip › Supplementary Figure 1.tif]
